# Secondary Germline Finding in Liquid Biopsy of a Deceased Patient; Case Report and Review of the Literature

**DOI:** 10.3389/fonc.2018.00259

**Published:** 2018-07-09

**Authors:** Maedah Veyseh, Charite Ricker, Carin Espenschied, Victoria Raymond, Anishka D’Souza, Afsaneh Barzi

**Affiliations:** ^1^Medicine, Keck School of Medicine of USC, University of Southern California, Los Angeles, CA, United States; ^2^Guardant Health, Inc., Redwood City, CA, United States

**Keywords:** cell-free DNA, germline mutation, liquid biopsy, hereditary cancer syndromes, pancreaticobiliary neoplasms

## Abstract

Liquid biopsies are increasingly used in the care of patients with advanced cancers. These tests are used to find mutations and other genomic alterations, quantify these findings over time, and guide treatment. It is not unexpected that germline mutations contributing to the development of cancer can be identified in cell-free DNA. Consequently, increased use of liquid biopsies has resulted in subsequent rise of secondary identification of germline mutations. Clinicians need to be aware of this potential use of liquid biopsies and the need to evaluate the patient and family members for confirmation. Our case documents a deceased patient’s liquid biopsy result that was confirmed as a germline mutation through a methodical work-up of the patient’s family members. Here, we present the case and provide a brief review of pertinent literature.

## Background

Tissue biopsies have been the gold standard for diagnosis in the field of oncology. Traditionally, a single biopsy was obtained to establish the cancer diagnosis and was primarily focused on understanding the site of cancer. With advancements in the understanding of the role of DNA alterations as a primary driver of tumorigenesis, the initial biopsy is now also used to assess the genomic alterations inherent to the tumor and ultimately to help guide therapeutic decision-making. However, the heterogeneous nature of cancer limits the ability to capture the spatial and temporal heterogeneity in a single baseline biopsy (1). Liquid biopsies, utilizing plasma derived cell-free circulating tumor DNA (cfDNA) have the ability to identify tumor derived somatic alterations with high concordance to tissue biopsy, similar patient outcomes as those with tumor identified somatic alterations and have the added advantage of being minimally invasive with the ability to capture evolving intra- and inter-tumoral mutations in patients with metastatic disease (2, 3). Given these features, cfDNA is increasingly being used to guide the use of targeted treatments in patients with newly diagnosed advanced cancers and those progressing on targeted therapies who may have developed resistance to therapy (4–7). Emerging areas of clinical use and active areas of research include: the utilization of cfDNA as an alternative cancer biomarker of tumor burden, to monitor disease progression, to detect metastasis, and to monitor response to therapy (8, 9).

Comprehensive cfDNA analysis utilizes next generation sequencing (NGS) to sequence both normal circulating leukocytic DNA, as well as the small proportion of cfDNA that is tumor derived.

The differentiation between somatic and germline mutations has been studied in tissue-based NGS (10, 11); however, there is limited published data on secondary germline findings of cfDNA by liquid biopsy.

Here, we describe the case of now deceased male with pancreatobiliary carcinoma with a secondary identified *BRCA2* alteration in cfDNA (Guardant360^®^). The finding led to subsequent familial testing and to the identification of a familial hereditary breast and ovarian cancer syndrome (HBOC).

## Case

**Figure 1:**
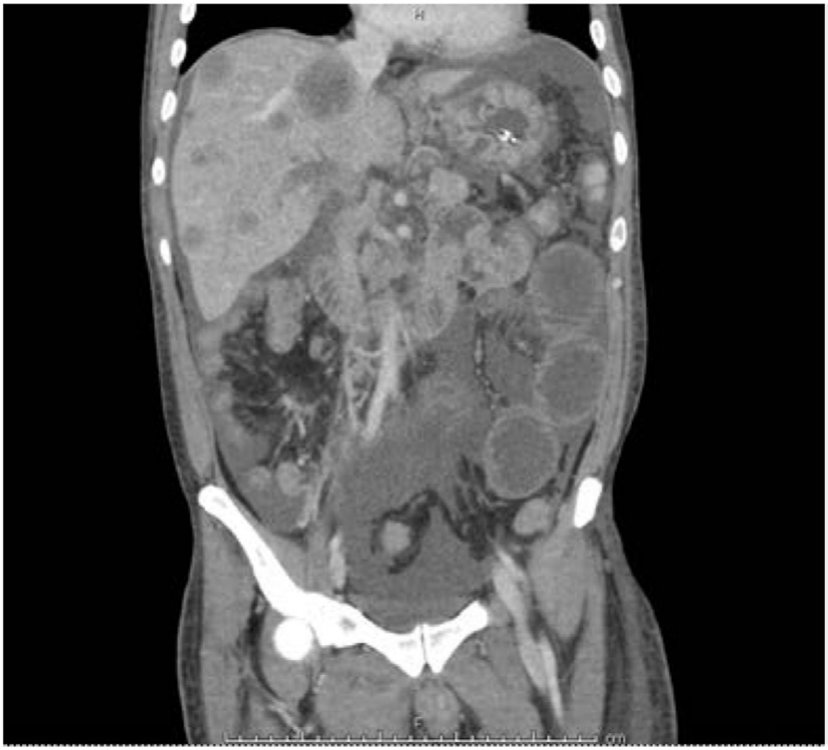
Frontal view panel showing liver metastasis and thickening of deuodenum and jejunum.

A 39-year-old Hispanic male of Salvadoran ancestry and no significant past medical history and a nonspecific family history of cancer, presented to the hospital with epigastric abdominal pain, nausea, and vomiting. Abdominal ultrasound showed multiple hypoechoic hepatic masses measuring up to 4.5 centimeters (cm) and the appearance favored metastatic disease. A follow-up computed tomography scan of chest, abdomen, and pelvis showed bilateral pulmonary embolus, retroperitoneal lymphadenopathy, and re-demonstration of the hepatic lesions (Figure 1). The patient underwent an ultrasound-guided liver biopsy, with pathology showing moderately to poorly differentiated adenocarcinoma with immunohistochemical stains favoring pancreatobiliary origin. A subsequent esophagogastroduodenoscopy and colonoscopy identified no definite primary malignancy. Due to the small amount of tumor tissue obtained on biopsy, comprehensive cfDNA analysis (Guardant360) was ordered with the goal of finding a targetable therapeutic mutation.

Over the 2 weeks following his clinical evaluation, the patient’s symptoms worsened and he was re-admitted to the hospital for intractable nausea and vomiting, abdominal pain, and subjective fever and chills. Further workup showed no evidence of bowel obstruction; however, the findings were highly suspicious for ischemic enteritis due to tumor obstruction of the portal vein. Given patient’s extremely debilitated state and poor performance status with an ECOG of 3, he was deemed not to be a candidate for further systemic therapy. He was discharged to home on hospice care and died within a few days.

Guardant360 is a New York State Department of Health-approved comprehensive cfDNA NGS assay that evaluates tumor derived genomic alterations in up to 73 genes and is performed at Guardant Health (Redwood City, CA, USA), a CLIA certified, College of American Pathologists (CAP) accredited laboratory. The gene list was selected to prioritize the identification of genomic alterations that are actionable—therapeutically targetable for an approved or late stage therapy, prognostic or predictive of therapeutic response, or informative of the presence of tumor-derived cfDNA. Point mutations in 73 genes, small insertions and/or deletions (indels) in 23 genes, copy number amplifications (CNAs) in 18 genes, and fusions in six genes are evaluated. single-nucleotide variants (SNVs), indels, and fusions are reported with a corresponding mutant allele fraction (MAF), calculated as the percentage of calls at a specific genomic position that are mutant over those that are wild type or mutant [mutant/(mutant + wild type)]. The reportable range for SNVs, indels, fusions, and CNAs is ≥0.04, ≥0.02, ≥0.04, and ≥2.12 copies, respectively (12). The median (or 50th percentile) MAF across more than 5,000 clinical samples tested on Guardant360 is 0.39%.

cfDNA was resulted after the patient’s death and were notable for the following four alterations and their corresponding MAF: *BRCA2* R2520* (66.02%), *TP53* L344P (20.92%) and R337G (19.26%), and *MET* Y989fs (0.21%) (Table 1). The *BRCA2* MAF twofold higher than the *TP53* MAF and within the range suspicious for germline variants. This finding, in combination with the patient’s young age at cancer diagnosis and nonspecific maternal family history of an early onset abdominal malignancy, raised the suspicion for a hereditary cancer syndrome.

**Table 1:** Alterations identified in cfDNA in the patient.

Alteration	% cfDNA
*BRCA2* R2520*	66.02
*TP53* L344P	20.92
*TP53* R337G	19.26
*MET* Y989fs	0.21

*Represents a change from arginine to a stop codon at amino acid 2520.

The genetic counselor was contacted by the medical oncologist to discuss the identification of the potentially germline *BRCA2* alteration identified in cfDNA. ClinVar^1^ and PubMed^2^ were both searched to determine if this particular alteration had been previously identified in the literature as a pathogenic germline mutation. R2520* corresponds to dbSNP:rs80358981 and in the clinical literature is reported as c.7558C > T (p.Arg2520*) or as 7786 C > T (R2520X). This nonsense mutation is located in exon 15 of the *BRCA2* gene and creates a premature stop codon. It is classified as pathogenic in ClinVar by all reporting clinical laboratories as well as by ENIGMA curation (13). A literature review found multiple publications that included reports of families with this mutation (14–16) and confirmed clinical history of HBOC.

The patient’s medical oncologist reviewed the cfDNA results with the deceased patient’s wife and offered her a genetics consultation to further discuss the findings and their potential implications. The patient’s wife was very interested in obtaining more information and a consult was scheduled with the medical oncologist and genetic counselor. During this visit, a discussion was held as to the role of somatic and germline mutations in cancer etiology and subsequently the parents contacted the counselor within 2 weeks of the initial consultation, confirming that they would participate in a consultation. The genetic counselor met with both parents and expanded on the family history previously reported. The 62-year-old mother confirmed that her father (patient’s maternal grandfather) was diagnosed with and died of stomach cancer at age 49 and her mother (patient’s maternal grandmother) died at 85 with no personal history of cancer. The 70-year-old father reported a maternal uncle (patient’s paternal great-uncle) with prostate cancer, diagnosed at an unknown age, and several first cousins with colorectal and uterine cancers, at unknown ages of diagnosis. After genetic counseling and a discussion of the limits and benefits of genetic testing, both parents underwent clinical genetic testing utilizing a multi-gene panel (Invitae Corporation, San Francisco, CA, USA). In addition, both consented to an USC IRB approved cancer genetics registry (0S-12-4). All family members agreed to publication or presentation of the results for scientific purposes and their agreement is noted in their medical records. No mutations were identified on the father’s analysis, but the mother was found to carry the same *BRCA2* mutation (c.7558C > T; R2520*) identified on the patient’s cfDNA analysis, confirming the diagnosis of HBOC within the family.

Both parents, as well as the deceased patient’s wife, presented to review the results of the genetic testing. The patient’s mother is 62, with one ovary intact; so, the personal implications for cancer risk management and prevention were discussed. In addition, she has other adult children who each has 50% probability of having inherited the *BRCA2* mutation. A family member letter was provided to facilitate communication of the patient’s mother’s genetic test results to her offspring. The deceased patient’s wife was counseled that given her children’s current age, no testing was indicated, as it would not impact their care. However, once they are adults they should discuss testing with their health-care providers, as breast cancer risk management begins at age 25 for mutation-positive women.

## Discussions and Review of Literature

The potential of liquid biopsies to identify a germline mutation is significant and the impact of such detection will extend beyond the patient to their family members to serve as a mechanism for cancer prevention. To our knowledge, this is the first case report of germline testing in the family members of a deceased individual, in whom a secondary *BRCA2* alteration was identified by liquid biopsy. Secondary unexpected genetic findings, regardless of the indication for ordering this test is an important and novel issue. We hereby will discuss how to trace and recognize such findings as germline on liquid biopsy and emphasize on their value even if the patient is deceased.

Discovery of secondary pathogenic germline variants in tumor tissue testing, confirmed by parallel normal DNA testing, have been reported. These secondary findings were found in 4.3% (19 out of 439) of patients in a study by Seifert et al. (17); and in 2.3% of 1,000 cancer patients in 19 cancer-related genes (18). Jones et al. analyzed matched tumor and normal DNA and identified germline alterations in cancer-predisposing genes in 3% of patients with apparently sporadic cancers (19). The frequencies of such findings have never been reported in liquid biopsies.

In tumor tissue sequencing, distinction between somatic and germline mutations can be challenging. Tumor-only sequencing approaches can not definitively identify germline alterations in cancer-predisposing genes and lead to an additional 31 and 65% false-positive findings in targeted and exome analyses, respectively, including in potentially actionable genes (19). The study by Mandelker et al. in a breast cancer population (101 patients) showed tumor-only testing identified *BRCA1/2* alterations in approximately 40% of the patients, with a majority of these patients not having germline mutations. Conversely “subtraction” of germline from tumor DNA sequence would have disguised 59 germline *BRCA1/2* cases (20). These data suggest that combined matched tumor-normal sequencing analyses are essential for precise identification and interpretation of somatic and germline alterations and have important implications for the diagnostic and therapeutic management of cancer patients. The same confirmatory principle with normal DNA testing should be applied to secondary germline findings discovered by NGS. This highlights the significance of our case, as the germline finding was confirmed by testing of the parent’s normal DNA (as the patient was deceased by the time of this necessary investigation). However, there are often barriers to collect additional tissue, including cost, putting patients through additional invasive procedures, and potential ethical concerns (21).

Studies have shown that examining the MAF of a suspected germline variant identified on tumor tissue genomic testing can be helpful in differentiating germline versus somatic status in the absence of normal tissue sequencing (tumor only testing). Germline variants often occur at an MAF of around 50% (when heterozygous) or around 100% (when homozygous, or due to loss of heterozygosity). Somatic alterations are acquired after birth and usually have an MAF < 50% (22). While Funchain et al. did report a mean MAF of 51% in tumors for variants confirmed to be in the germline, the range of MAF was 35–72% (23). Meric-Bernstam et al. reported that the median MAF was higher for confirmed germline alterations compared to somatic alterations (46 versus 33%). However, the range of MAF for germline alterations was 13–94%, highlighting the importance of considering more than MAF when evaluating tumor alterations and their potential to be germline in origin.

Our literature search revealed several other studies on plasma cfDNA testing that also used MAF to identify secondary potentially germline findings. However, unlike our case, these studies used germline testing of the same individuals to confirm their findings (24, 25). Hu et al. reported a patient with metastatic lung adenocarcinoma and positive family history of lung cancer who had both *EGFR* L858R and *EGFR* T790M mutations on tissue NGS. Plasma NGS (Guardant360) detected initial MAF of *EGFR* L858R as 5.3%, fluctuating during the course of treatment; whereas initial MAF of T790M identified as 50.9%, stayed constant during therapy. The latter mutation’s trend and a positive family history raised the suspicion of an underlying germline mutation, which was verified by germline testing. The investigators then tested their theory on a large cohort of cancer patients (*n* = 31,414), showing that the MAF of *EGFR* T790M in plasma NGS samples can aid in differentiating germline and somatic alterations (24). Shukuya et al. reported a case with lung adenocarcinoma and no personal or family history of breast or ovarian cancer, who had a *BRCA2* mutation with MAF of 50.7% identified on cfDNA. An underlying germline mutation was suspected due to the MAF being at a frequency suspicious for germline alterations. Germline status was confirmed after referral to genetic counseling and germline testing (25). In a third study examining samples from over 10,000 patients, 1.7% (*n* = 173) of patients had a putative germline alteration identified on cfDNA, with the majority of these alterations having an MAF ranging between 40 and 55% (26).

The MAF of the *BRCA2* alteration reported in our case was 66%, nearly twofold higher than the cooccurring *TP53* alterations identified on the same sample. This relatively high MAF increased our suspicion that the *BRCA2* alteration was of germline in origin. Other findings that can be suggestive of an alteration being germline include finding a well-characterized mutation in a hereditary cancer predisposing gene, such as one of the known *BRCA* founder mutations. Another characteristic of a germline alteration is a relatively consistent MAF over sequential tests. In contrast, somatic alterations tend to fluctuate, as illustrated in the other case report referenced above (25, 27). While the alteration identified in our patient’s assay was not one of the *BRCA1/2* founder mutations, it is a mutation that is well-documented in the clinical literature as occurring in the germline setting and is known to be pathogenic. Our case is the only report that entertained MAF percentage and trend as a clue to track the same mutation in a patient’s close relation, as the patient’s own DNA was not available at that time to confirm this finding as germline. This was necessary to verify a hereditary malignancy present in this patient’s family and would have significant implications for his family members.

Based on the ACMG (American College of Medical Genetics and Genomics) recommendation for secondary findings in exome and genome sequencing, only known pathogenic or expected pathogenic variants should be reported to patients. The ACMG recommends that laboratories performing clinical sequencing, report pathogenic variants in 59 genes, regardless of the indication for testing (28). Schrader et al. suggested that there is a potential value to a broad germline sequencing approach in the context of tumor-normal analysis. In their study of 1,566 cancer patients, 16 were found to carry potentially pathogenic variants in known Mendelian disease-associated genes and 59% of the individuals with a potentially pathogenic variant in a cancer-susceptibility gene had cancer not known to be associated with that gene (29). A Joint Consensus Recommendation from the Association for Molecular Pathology, American Society of Clinical Oncology, and CAP published in 2016 spoke to secondary germline findings identified in the course of tumor testing, recommending that germline variations with evidence of clinical impact be reported (22). We suggest the same principles applied to exome and genome sequencing, as well as other tumor sequencing, be translated to plasma genotyping as well. As outlined in Robson et al., oncologists ordering somatic genomic tumor testing should counsel their patients about the potential to identify secondary findings outside of the primary indication for testing (30). Our case highlights that patients undergoing cfDNA tumor analysis should be counseled similarly due to the potential to identify underlying germline alterations. Ordering clinicians should consider that it may not be possible for patients to “opt out” of learning germline mutation status (e.g., a *BRCA2* alteration may be germline, and also makes patient eligible for treatment with PARP inhibitors, making the genomic finding important for therapeutic decision-making—the primary indication for ordering somatic tumor testing). Also, given that most somatic tumor testing, utilizing tumor tissue or cfDNA, is being performed in patients with late stage cancers, ordering clinicians should have a discussion with their patients about alternative individuals to whom potential germline results can be returned, as evidenced by our experience. Finally, ordering clinicians should be aware of resources within or near their institution to help with interpretation of potential germline alterations identified on somatic tumor testing, and genetic counseling resources available for their patients (27).

Incorporating tumor genomic information into a patient’s therapeutic decision-making is the premise of precision oncology (31). We expand on that premise and suggest that precision oncology can provide a mechanism for identification of families appropriate for genetic counseling and cancer prevention. Clinicians should be attentive to the potential to identify secondary germline alterations, as they can have great therapeutic and preventive implications for patients and their families. With increased use of liquid biopsies in clinical practice to help with treatment decisions and to offer targeted therapies, development of an algorithm for identification and confirmation of potential germline mutations identified through this testing is critical. Clinicians should consider the personal and family history of the patient, along with information in the cfDNA results including reported pathogenicity of the variant in the clinical literature, MAF of the variant, and MAF relative to other variants identified on the sample. Strategies should be put in place for genetic counseling referral in case of such discoveries. A standard method of analysis and interpretation of these test results is essential to prevent any lost opportunity for prevention in the patient and their at-risk family members.

## Author Contributions

Reviewed clinical data, performed literature review, and wrote majority of paper.

## Conflict of Interest Statement

The authors declare that the research was conducted in the absence of any commercial or financial relationships that could be construed as a potential conflict of interest. The reviewers, AS and CB, and handling Editor declared their shared affiliation.

## Footnotes

^1^www.ncbi.nlm.nih.gov/clinvar/ (Accessed: December 7, 2017).

^2^www.ncbi.nlm.nih.gov/pubmed/ (Accessed: December 7, 2017).
