# Supplementary material for: Secondary Germline Finding in Liquid Biopsy of a Deceased Patient; Case Report and Review of the Literature
Source: Front Oncol. 2018 Jul 9;8:259. doi: 10.3389/fonc.2018.00259 (PMC6052887; doi:10.3389/fonc.2018.00259)
Supplement: Supplementary file 1 [file table_1.docx]

Supplementary Data

Table 1: Genes include in parental assays

1. **Mother:**

| Genes evaluated for sequence changes and exonix deletions/duplications | *ALK, APC, ATM, AXIN2, BAP1, BARD1, BLM, BMPR1A, BRCA1, BRCA2, BRIP1, CASR, CDC73, CDH1, CDK4, CDKN1B, CDKN1C, CDKN2A (p14ARF), CDKN2A (p16INK4a), CEBPA, CHEK2, CTNNA1, DICER1, DIS3L2, EPCAM* (deletion/duplication testing only), *FANCC, FH, FLCN, GATA2, GPC3, GREM1* (promoter region deletion/duplication testing only), *HRAS, KIT, MAX, MEN1,*  *MET, MLH1, MSH2, MSH6, MUTYH, NBN, NF1, NF2, PALB2, PALLD, PDGFRA, PHOX2B, PMS2, POLD1, POLE, POT1, PRKAR1A, PTCH1, PTEN, RAD50, RAD51C, RAD51D, RB1, RECQL4, RET, RUNX1, SDHAF2, SDHB, SDHC, SDHD, SMAD4, SMARCA4, SMARCB1, SMARCE1, STK11, SUFU, TERC, TERT, TMEM127, TP53, TSC1, TSC2, VHL, WRN, WT1* |
| --- | --- |
| Genes evaluated for sequence changes only | *EGFR* (c.2369C>T, p.Thr790Met variant only), *HOXB13* (c.251G>A, p.Gly84Glu variant only), *MITF* (c.952G>A, p.Glu318Lys variant only), *SDHA* |

1. **Father:**

| Genes evaluated for sequence changes and exonix deletions/duplications | *AKT1, ALK, APC, ATM, AXIN2, BAP1, BARD1, BLM, BMPR1A, BRCA1, BRCA2, BRIP1, BUB1B, CASR, CDC73, CDH1, CDK4, CDKN1B, CDKN1C, CDKN2A (p14ARF), CDKN2A (p16INK4a), CEBPA, CHEK2, CTNNA1, DICER1, DIS3L2, ENG, EPCAM* (deletion/duplication testing only), *FAM175A, FANCC, FH, FLCN, GALNT12, GATA2, GPC3, GREM1* (promoter region deletion/duplication testing only), *HRAS, KIT, MAX, MEN1, MET, MLH1, MLH3, MRE11, MSH2, MSH6, MUTYH, NBN, NF1, NF2, PALB2, PALLD, PDGFRA, PHOX2B, PIK3CA, PMS2, POLD1, POLE, POT1, PRKAR1A, PTCH1, PTEN, RAD50, RAD51C, RAD51D, RB1, RECQL4, RET, RINT1, RUNX1, SDHAF2, SDHB, SDHC, SDHD, SMAD4, SMARCA4, SMARCB1, SMARCE1, STK11, SUFU, TERC, TERT, TMEM127, TP53, TSC1, TSC2, VHL, WRN, WT1, XRCC2* |
| --- | --- |
| Genes evaluated for sequence changes only | *EGFR* (c.2369C>T, p.Thr790Met variant only), *HOXB13* (c.251G>A, p.Gly84Glu variant only), *MITF* (c.952G>A, p.Glu318Lys variant only), *SDHA* |
